# Effect of gene *CRTC2* on the differentiation of subcutaneous precursor adipocytes in goats

**DOI:** 10.5713/ab.24.0248

**Published:** 2024-10-28

**Authors:** Xuening Li, Tingting Hu, Ruiwen Li, Yanyan Li, Yaqiu Lin, Yong Wang, Wei Liu, Youli Wang

**Affiliations:** 1 Key Laboratory of Qinghai-Tibetan Plateau Animal Genetic Resource Reservation and Utilization of Education Ministry, Southwest Minzu University, Chengdu, China; 2 Key Laboratory of Qinghai-Tibetan Plateau Animal Genetic Resource Reservation and Exploitation of Sichuan Province, Southwest Minzu University, Chengdu, China; 3 College of Animal and Veterinary Science, Southwest Minzu University, Chengdu, China; 4 Chengdu Women’s and Children’s Central Hospital, School of Medicine, University of Electronic Science and Technology of China, Chengdu, China

**Keywords:** Adipogenesis, Biological Property, *CRTC2*, Goat, Subcutaneous Precursor Adipocytes

## Abstract

**Objective:**

The aim of this study was to obtain goat *CRTC2* gene sequence and elucidate its biological properties, and further study the impact of overexpression and interference of *CRTC2* on the cell differentiation of goat subcutaneous precursor adipocytes.

**Methods:**

The sequence of goat *CRTC2* was cloned by reverse transcription (RT)-polymerase chain reaction (PCR) and its molecular characterization was analyzed. The expression of *CRTC2* gene in goat tissues and subcutaneous precursor adipocytes differentiated from 0 to 120 h was examined by quantitative real-time PCR (qRT-PCR). The effects of *CRTC2* on the subcutaneous precursor adipocyte differentiation were investigated by using liposome transfection, Bodipy, Oil Red O staining and qPCR.

**Results:**

The results showed that the cloned goat *CRTC2* gene was 2363 bp long (coding sequence [CDS] 2082 bp), encoding 693 amino acids. The relative expression levels of *CRTC2* gene were highest in liver and then in kidney (*p*<0.05). During differentiation, the highest expression of *CRTC2* in subcutaneous precursor adipocytes was observed at 120 of differentiating (*p*<0.01). In addition, we found that overexpression of *CRTC2* significantly increased the expression of lipid metabolism-related genes (*C/EBPα*, *C/EBPβ*, *PPARγ*, *DGAT1*, *DGAT2*, *ACC*, *FASN*, *SREBP1*, *AP2*, *LPL*, *ATGL*) and promoted lipid accumulation. We then chemically synthesized goat *CRTC2* small interfering RNA and transfected it into goat subcutaneous precursor adipocytes. The results revealed that SiRNA-mediated interference with *CRTC2* significantly inhibited its differentiation and suppressed lipid droplet aggregation.

**Conclusion:**

So, this study indicates that *CRTC2* is a positive regulator that promoting cell differentiation of subcutaneous adipocyte in goats, which lays the foundation for an in-depth study of the role of *CRTC2* in lipid deposition in goats.

## INTRODUCTION

Adipose tissue is an active endocrine organ that involved in a variety of activities such as insulin sensitivity, glucose tolerance, lipid metabolism and deposition [1,2]. It has been found that adipose is distributed throughout the body of goat, including subcutaneous, visceral, intramuscular, intermuscular and caudal. Subcutaneous adipose tissue is the most widely distributed adipose and is closely related to carcass characteristics such as intramuscular fat content, tenderness, and flavor [3–5]. Similar to the role of intermuscular and intramuscular fat in meat characteristics, subcutaneous adipose tissue affects the texture, flavor and nutritional value of edible meat [6]. Therefore, it is important to reveal the metabolic characteristics and regulatory mechanisms of subcutaneous fat development.

CREB-regulated transcriptional co-activators (CRTCs) are a class of transcriptional co-activators that promote the transcriptional activity of basic leucine zipper-type transcription factors, including CREBs [7,8]. There are three family members of CRTCs, including *CRTC1*, *CRTC2*, and *CRTC3*. Prior studies have shown that *CRTC1* is expressed predominantly in the brain and is involved in brain synaptic plasticity, learning, long-term memory formation and mitochondrial metabolic activities [9]. *CRTC3* is highly expressed in adipose tissue and plays an important role in insulin resistance and energy metabolism [10]. Differently, *CRTC2* is widely expressed in most peripheral tissues, such as muscle [11] and liver [12]. It has been suggested that *CRTC2* is responsible for the transcriptional regulation of hepatic gluconeogenesis [13], and the role of *CRTC2* in hepatic lipid metabolism has recently been elucidated [14]. In addition to its role as a transcriptional co-activator, *CRTC2* has been shown to interact with the coat protein complex II subunit protein Sec31A to inhibit sterol regulatory element-binding protein-1 (*SREBP1*) mediated lipogenesis in the liver [15]. Besides, induction of cholesterol synthesis was observed through *CRTC2*/*FOXO1*-mediated transcriptional activation of *SREBP2* [16], suggesting that *CRTC2* is a key protein in the regulation of hepatic glucose and lipid metabolism. Hepatic-specific *CRTC2* was found to have no significant changes in hepatic lipid metabolism in normal diet-fed mice, but metabolic homeostasis was altered in hepatic *CRTC2*-deficient high-fat diet mice [17]. Currently, it has been shown that this gene plays an important role in many physiological activities such as lipid metabolism in Nile tilapia [18], rat [19], and cattle [20]. However, the effect of *CRTC2* gene on subcutaneous precursor adipocyte differentiation and lipid metabolism in goats is still unclear.

This study intends to investigate the effect of *CRTC2* on adipogenesis in subcutaneous precursor adipocytes of Jianzhou Daer goats. First, we cloned the sequence of goat *CRTC2* gene by using reverse transcription (RT)-polymerase chain reaction (PCR) technology, and carried out bioinformatics analysis, then we used real-time fluorescence quantitative PCR (qPCR) technology to study its expression level in various tissues of the goat, and constructed tissue expression profiles and cellular temporal expression profiles, and further investigated the effects of overexpression and interference of *CRTC2* on the differentiation of goat subcutaneous precursor adipocytes was further investigated by overexpression and interference. The results of this study will provide important basic data for the final elucidation of the mechanism of *CRTC2* regulation of subcutaneous precursor adipocyte differentiation in goats, and provide a new strategy for molecular breeding of goats.

## MATERIALS AND METHODS

### Sample collection

All animal experiments were reviewed by Animal Experimental Ethical Inspection of Southwest University for Nationalities (No.2020086). In this experiment, seven-day-old Jianzhou Daer goats (n = 3) were purchased from Sichuan Tiandi Goat Biological Engineering Co., Ltd. (Chengdu, China). After slaughter, heart, liver, spleen, lung, kidney, week of kidney fat, longissimus dorsi muscle and small intestine tissue samples were collected. Then were washed with phosphate buffered saline (PBS), wrapped in tin foil and placed in cryopreservation tubes. Samples were immediately frozen in liquid nitrogen for later analysis.

### Cell isolating and culturing

The primary cells for this experiment were obtained from subcutaneous adipocytes of 7-day-old Jianzhou Daer goats and were preserved in liquid nitrogen in the laboratory. Goat subcutaneous preadipocytes isolation and culture methods were in accordance with previously described methods [21]. Briefly, subcutaneous adipose tissue was isolated from healthy 7-day-old Jianzhou Daer goats under aseptic conditions, and connective tissue and blood vessels were removed. After rinsing 2 to 3 times with antibiotic-containing PBS, the subcutaneous adipose tissue of the goats was cut with scissors and digested with type I collagenase (Sigma, Shanghai, China) for 1 h at 37°C. The cell suspension was then centrifuged again at 1,500 r·min^−1^ for 5 min and the cells were removed from the cells. The cell suspension was then centrifuged again at 1,500 r·min^−1^ for 5 min, and the preadipocytes were resuspended in Dulbecco’s modified essential medium (DMEM)/F12 (Hyclone, Logan, UT, USA) containing 10% fetal bovine serum (FBS) (Gemini, Calabasas, CA, USA) and 1% penicillin-streptomycin (Gemini). Finally, the cell suspension was transferred to cell culture flasks and cultured in an incubator at 37°C and 5% CO_2_ to obtain primary subcutaneous adipocytes.

### Cloning of goat *CRTC2* gene

Based on the predicted sequence of goat *CRTC2* (GeneID: 102180907) on NCBI, primers were designed using Primer Premier 5.0 software (Table 1) and synthesized by Beijing Qingke Biotechnology Co., Ltd. the PCR reaction system consisted of 25 μL of Primer STAR Max DNA Polymerase (TaKaRa, Tokyo, Japan). The PCR reaction system consisted of 25 μL of Primer STAR Max DNA Polymerase, 2.0 μL (10 μmol L^−1^) of positive and negative primers, 2.0 μL of goat small intestine cDNA and 19 μL of ddH_2_O. The reaction conditions were as follows: PCR amplification program: pre-denaturation (98°C, 2 min); denaturation (98°C, 10 s), annealing (60°C, 15 s), extension (72°C, 45 s), 35 cycles; extension (72°C, 2 min), 4°C holding. Amplification products were recovered by agarose gel electrophoresis and ligated with 007 VS cloning vector (Qingke, Beijing, China) for 5 min at 25°C in a metal bath and transformed into E. coli DH5α competent cells (Qingke). DH5α competent cells, take appropriate amount of bacterial solution and apply it to the plate containing ampicillin (Biosharp, Shanghai, China), place it in 37°C constant temperature incubator and incubate overnight, pick the monoclonal colonies to expand the culture for 10 h, and then send the bacterial solution (at least 3 tubes) identified as positive by PCR to Qingke Bio-technology for sequencing. Bioinformatics analyses are shown in Table 2.

### Vectors construction, chemical synthesis of siRNA, and transfection

*EcoR* I and *Xho* I (TaKaRa) cleavage sites were selected and the goat *CRTC2* expression vector was constructed by double cleavage. The pEGFP-N1 plasmid and the subcloned products were double digested separately to purify the fragments. The fragments were ligated with T4 ligase (TaKaRa) at 16°C overnight. After confirmation of digestion, the fragments were transformed into DH5α to screen positive colonies for sequencing. The correctly sequenced bacterial fluids were amplified and the plasmids extracted (Supplement 1). In RNA interference experiments, Gene Pharma (Shanghai, China) designed and synthesized SiRNA targeting *CRTC2* (named Si-*CRTC2*) and negative control SiRNA (named Si-NC). All transfection experiments were performed using TurboFect (Thermo Fisher Scientific, Waltham, MA, USA) according to the manufacturer’s instructions. For example, 6-well plates were transfected with 2 μg of plasmid DNA per well at 70% to 80% cell confluence. To silence *CRTC2*, 2 μg of Si-*CRTC2* or Si-NC was used in each well. The solution was changed after 16 h and cells were collected for subsequent experiments after 48 h of incubation.

### Induced differentiation of goat subcutaneous preadipocytes

For differentiation of goat subcutaneous preadipocytes, culture goat subcutaneous preadipocytes in DMEM/F12 cell culture medium (containing 10% fetal bovine serum and 1% antibiotics). For example, third-generation goat subcutaneous preadipocytes were inoculated into 6-well plates with 8×10^4^ cells per well. After 16 h of transfection, the cells were cultured in adipocyte induction medium (DMEM/F12 with 10% FBS, 1% antibiotics and 50 μmol L^−1^ oleic acid (Sigma-Aldrich, St. Louis, MO, USA) for 48 h.

### Oil Red O staining, Bodipy staining and 4′,6-diamidino-2-phenylindole staining

Cells for staining were grown in 24-well plates. The transfection system corresponded to half of a 12-well plate. After 48 h of induction, the medium was discarded, washed three times with PBS and fixed with 4% paraformaldehyde for 30 min at room temperature. For Oil Red O staining, 200 μL of Oil Red O working solution (Oil Red O stock solution : ddH_2_O = 3:2) (Solarbio, Beijing, China) was added to each well and the red lipid droplets were observed after 30 min of staining. After staining and washing, the cells were observed and photographed using an Olympus IX-73 fluorescence microscope (Olympus, Tokyo, Japan). For quantification of Oil Red O, 1 mL of 100% isopropanol was added to each well to extract the dye. The absorbance of the extracted dye at 490 nm was then detected using an enzyme marker.

Bodipy and 4′,6-diamidino-2-phenylindole (DAPI) staining were performed. Cells were washed three times with PBS, and then Bodipy working solution (Bodipy stock solution : PBS = 1:1,000, 200 μL per well) purchased from Thermo Fisher Scientific was added to each well of the plate under the condition of avoiding light, and the staining was avoided from light for 10 min, then washed with PBS, and DAPI (DAPI stock solution : PBS = 1:1,000) dye was added for 10 min. DAPI (DAPI stock solution : PBS = 1:1,000, 200 μL per well) was added to each plate well, and then washed with PBS after 10 min of light-avoidance staining. Finally, the aggregation of lipid droplets in adipocytes was observed under a fluorescence microscope and photographed.

### RNA isolation and quantitative reverse transcription polymerase chain reaction analysis

Extract total RNA from goat tissues using the Trizol reagent (TaKaRa) method, and reverse transcribe the RNA into cDNA using the Revert Aid MM kit (Thermo Fisher Scientific). The ubiquitin-expressing transcript (*UXT*) gene was individually selected as an internal reference gene to normalize mRNA levels. The relative expression level of *CRTC2* gene in subcutaneous adipocytes was detected by qPCR technique. Relative expression levels of *CRTC2* gene in subcutaneous adipocytes. The relative expression levels of *CRTC2* gene in subcutaneous adipocytes at different different differentiation times (0, 12, 24, 36, 48, 60, 72, 84, 96, 108, 120 h). Total PCR system 20 μL (premix 2× TBG 10 μL, ddH_2_O 6 μL, upstream 1 μL, downstream 1 μL and cDNA 2 μL). PCR process 40 cycles of pre-denaturation at 95°C for 3 min, denaturation at 94°C for 10 sec, annealing at 60°C for 20 sec and extension at 72°C for 30 sec. When detecting the effects of overexpression of *CRTC2* and Si-*CRTC2* on the differentiation of subcutaneous adipocytes in goats, adipogenic genes (peroxisome proliferator-activated receptor γ [*PPARγ*], CAAT enhancer binding protein α [*C/EBPα*], CAAT enhancer binding protein β [*C/EBPβ*], triglycerides (TG) synthesis genes (diacylglycerol acyltransferase 1 [*DGAT1*], *DGAT2*, acetyl coenzyme A carboxylase [*ACC*], fatty acid synthase [*FASN*], *SREBP1*, fatty acids binding protein [*AP2*]), as well as TG lipolysis genes (lipoprotein lipase [*LPL*], adipose triglyceride lipase [*ATGL*]) were used. Specific primer sequences were designed as Table 1. qRT-PCR was carried out with a BioRad Real-Time PCR system (Hercules, CA, USA).

### Western blot analysis

Cells in the cell plate were rinsed twice with PBS and then lysed with RIPA buffer containing protease inhibitors. The protein concentration of each sample was determined and denatured at 100°C according to the instructions of the BCA Protein Assay Kit (Biosharp). Western blotting analyses were performed as previously described in the literature [22]. Protein lysates (20 μg/lane) were detected on 12% sodium dodecyl sulfate polyacrylamide gel electrophoresis gels and transferred to polyvinylidene difluoride membranes. The membranes were then immersed in 5% skimmed milk for 2 h at room temperature, followed by incubation with anti-CRTC2 (1:500; PTM BIO, Hangzhou, China), anti-C/EBPα (1:1000; Cell Signaling, Danvers, MA, USA), and anti-β-actin (1:1,000; Cell Signaling) at 4°C overnight. The membranes were incubated with horseradish peroxidase-conjugated secondary antibody (1:5,000; Invitrogen, Waltham, MA, USA) for 1 h at room temperature. Finally, we detected the target proteins using enhanced chemiluminescence (Bio-Rad).

### Statistical analysis

The qRT-PCR data were analyzed using the 2^−ΔΔCt^ method, with qRT-PCR data presented as mean±standard error, and the significance of the data was analyzed using one-way analysis of variance (ANOVA) and t-tests, and significance of more than two groups were analyzed using multiple comparison tests. Statistical significance was considered when p<0.05. “*” indicates a significant difference (p<0.05) and “**” indicates a highly significant difference (p<0.01). The qRT-PCR results were plotted using GraphPad Prism 9.5.1.

## RESULTS

### Cloning of goat *CRTC2* gene

**Figure 1:**
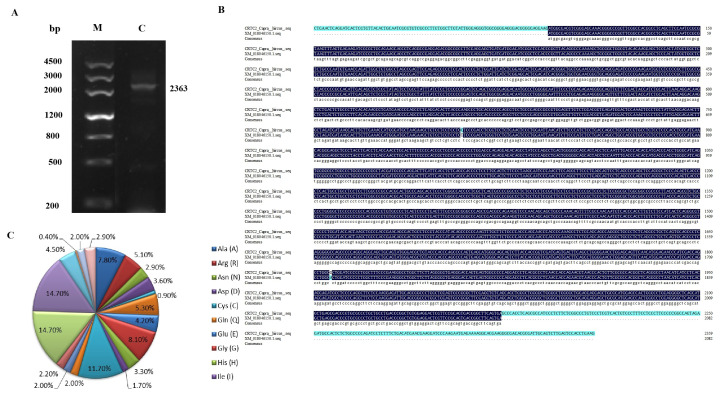
Sequence analysis of the goat *CRTC2* gene. (A) Goat *CRTC2* gene amplification results. (B) *CRTC2* gene sequence alignment with predicted sequence. (C) Amino acid composition of CRTC2 protein.

PCR amplification yielded fragments of the expected target product size (Figure 1A). The goat *CRTC2* gene is 2363 bp in length, of which the complete coding sequence (CDS) region is 2082 bp, which is consistent with the predicted sequence (XM_018046150.1). In contrast, the CDS region has two base mutations. Comparison revealed that the 807th locus has T>C and A>G at position 1806, but did not affect protein translation and were synonymous mutations (Figure 1B). The goat *CRTC2* gene encodes a total of 693 amino acids, of which 102 are proline (Pro) and serine (Ser) in the highest proportion (14.7%), followed by 81 leucine (Leu, 11.7%, Figure 1C).

### Bioinformatics analysis of the *CRTC2* gene in goats

**Figure 2:**
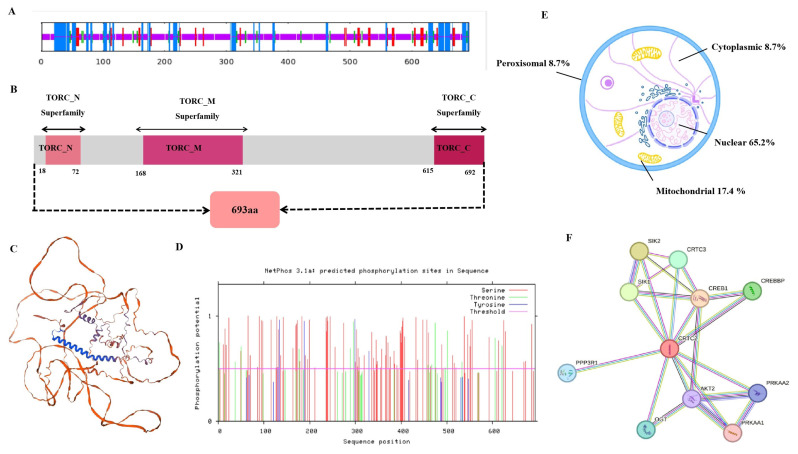
Bioinformatics analysis of the *CRTC2* gene in goats. (A) Secondary-structure prediction. (B) Functional structure domain prediction. (C) Tertiary-structure prediction. (D) CRTC2 protein phosphorylation site prediction. (E) Subcellular localization. (F) Interaction protein prediction of goat *CRTC2*.

The secondary structure of *CRTC2* was predicted using SOPMA software, and the structural composition of the protein had 18.47% (128 amino acids) of the total amino acids in α-helices, 7.79% (54 amino acids) in extended strands, 68.25% (473 amino acids) in random coils, and 5.48% (38 amino acids) in β-turns (Figure 2A). The amino acid sequence was analyzed using InterPro online software to predict the functional structural domains of *CRTC2*, and the protein was found to have three protein structural domains, TORC_N, TORC_M, and TORC_C, which are located at sites 18–72, 168–321, and 615–692 of the entire sequence (Figure 2B). The tertiary structure of *CRTC2* was predicted using SWISS-MODEL, which was consistent with the secondary structure prediction (Figure 2C); The physicochemical properties of goat CRTC2 protein were analyzed using SOPMA software. The results showed that the molecular weight of goat CRTC2 protein was 73 kDa. The theoretical isoelectric point was 6.54, the instability coefficient was 82.41, and the Grand average of hydropathicity was -0.516. It was presumed that goat CRTC2 protein was an unstable hydrophilic acidic protein. Since there are more negatively charged amino acids than positively charged amino acids, the goat CRTC2 protein is negatively charged. The goat CRTC2 protein has 107 potential phosphorylation sites, including 88 serine (Ser) phosphorylation sites, 14 threonine (Thr) phosphorylation sites and 5 tyrosine (Tyr) phosphorylation sites (Figure 2D). Subcellular localisation predicts that CRTC2 protein is mostly distributed in the nucleus (73.9%), followed by vesicles (4.3%), cytoskeleton nucleus (65.2%), followed by mitochondria (17.4%), peroxisomes (8.7%), and cytoplasm (8.7%, Figure 2E). Prediction of CRTC2-interacting proteins by the STRING database showed that goat CRTC2 interacted strongly with proteins such as CRTC3, AKT2, CREB1, CREB2, SIK1 and SIK2 (Figure 2F).

### Tissue and temporal expression analysis of goat *CRTC2* gene

**Figure 3:**
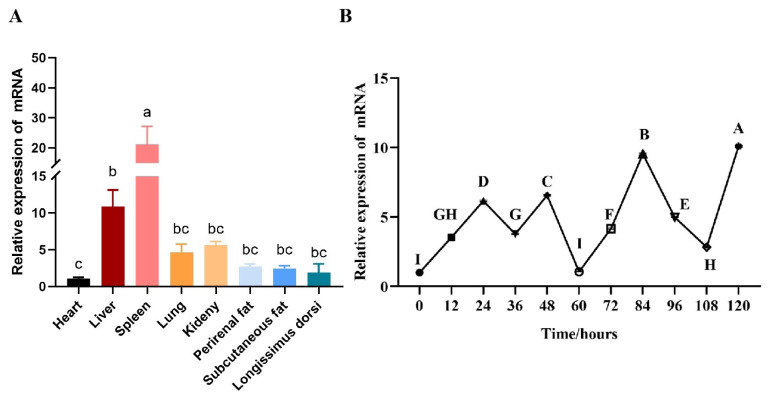
Tissue and temporal expression analysis of goat *CRTC2* gene. (A) Distribution of *CRTC2* in different tissues of goats. (B) Temporal expression profile of goat *CRTC2* gene in subcutaneous precursor adipocytes. ^a–c^ p<0.05, ^A–I^ p<0.01.

The results were compared with the expression level of heart. The results of qRT-PCR showed that the expression of *CRTC2* gene was higher in the liver tissue of goats (p<0.05), followed by the kidney (p<0.05, Figure 3A). Different uppercase letters in the figure indicate highly significant differences (p<0.01), and different lowercase letters indicate significant differences (p<0.05), as below. The goat *CRTC2* gene was differentially expressed from 0 to 120 h of subcutaneous precursor adipocyte differentiation, and the relative expression was up-regulated in all cases. The difference in up-regulation was not significant at 60 h compared to 0 h when cells were undifferentiated, and the expression level of *CRTC2* was highly significantly up-regulated for the rest of the time period (p<0.01, Figure 3B).

### Overexpression of *CRTC2* promotes the differentiation of subcutaneous precursor adipocytes in goats

**Figure 4:**
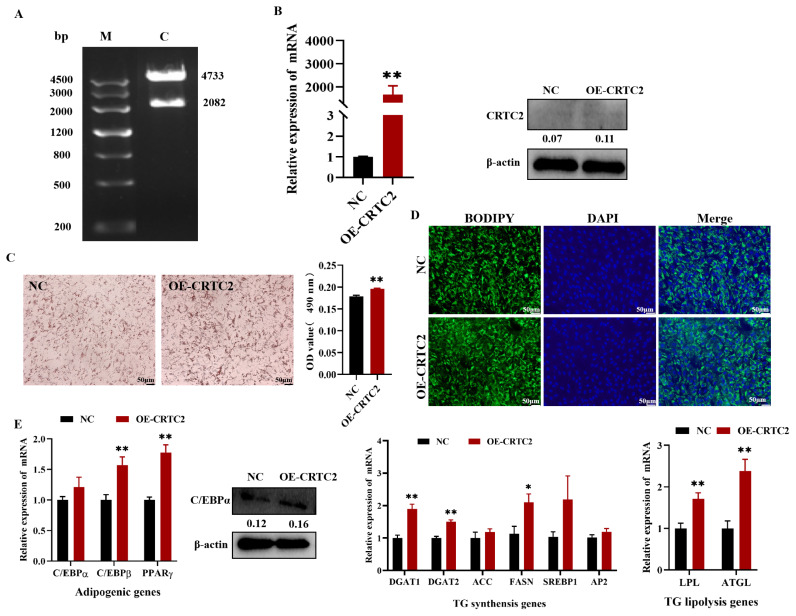
Effect of overexpression of goat *CRTC2* on adipocyte differentiation in subcutaneous precursor adipocytes. (A) Effect of overexpression of pEGFP-*CRTC2* double enzyme digest product. (B) qRT-PCR and protein level image of overexpressing *CRTC2* efficiency. (C) Oil red O staining and Semi-quantitative evaluation of absorbance assay of Oil Red O content performed at 490 nm. (D) Bodipy staining. (E) Effects of overexpression of *CRTC2* on expression of lipid metabolism marker genes. * p<0.05, ** p<0.01. DAPI, 4′,6-diamidino-2-phenylindole; TG, triglycerides; qRT-PCR, quantitative reverse transcription polymerase chain reaction.

Agarose gel electrophoresis showed two bands of pEGFP-N1 (4733 bp) and *CRTC2* (2082 bp) that were in line with the expected results, consistent with the sequencing results, indicating that the overexpression vector was successfully constructed (Figure 4A). The constructed pEGFP-N1-*CRTC2* (named OE-*CRTC2*) or pEGFP-N1 (named NC) plasmids were transfected into goat subcutaneous precursor adipocytes for functional studies. *CRTC2* expression was up-regulated approximately 1600-fold (p<0.01) as detected by qPCR, and western blot (WB) also verified the overexpression efficiency of *CRTC2* in goat intramuscular precursor adipocytes (Figure 4B; Supplement 2). The formation of intracellular lipid droplets was visualized by Bodipy, DAPI and Oil Red O staining, and it was found that overexpression of *CRTC2* promoted the subcutaneous adipocytes. The accumulation of intracellular lipid droplets was observed by Bodipy and Oil Red O staining (Figures 4C, 4D). The results of lysing the two groups of cells with isopropanol and detecting their absorbance values at 490 nm showed that the OD490 value of OE-*CRTC2* was highly and significantly up-regulated compared with that of the NC group (p<0.01). To investigate the changes in the expression of lipid metabolism-related genes induced by *CRTC2* overexpression, we assessed the expression of these genes in *CRTC2* overexpressing cells (Figure 4E; Supplement 2). *CRTC2* expression caused significant increases in the adipogenesis genes *C/EBPβ* and *PPARγ* (both p<0.01), the TG synthesis genes *DGAT1*, *DGAT2* (both p<0.01), *FASN* (p<0.05) and TG catabolic genes *LPL* and *ATGL* (both p<0.01) were significantly increased. These data suggest that overexpression of *CRTC2* promotes lipid accumulation in goat subcutaneous precursor adipocytes.

### Si-*CRTC2* inhibits the differentiation of subcutaneous precursor adipocytes in goats

**Figure 5:**
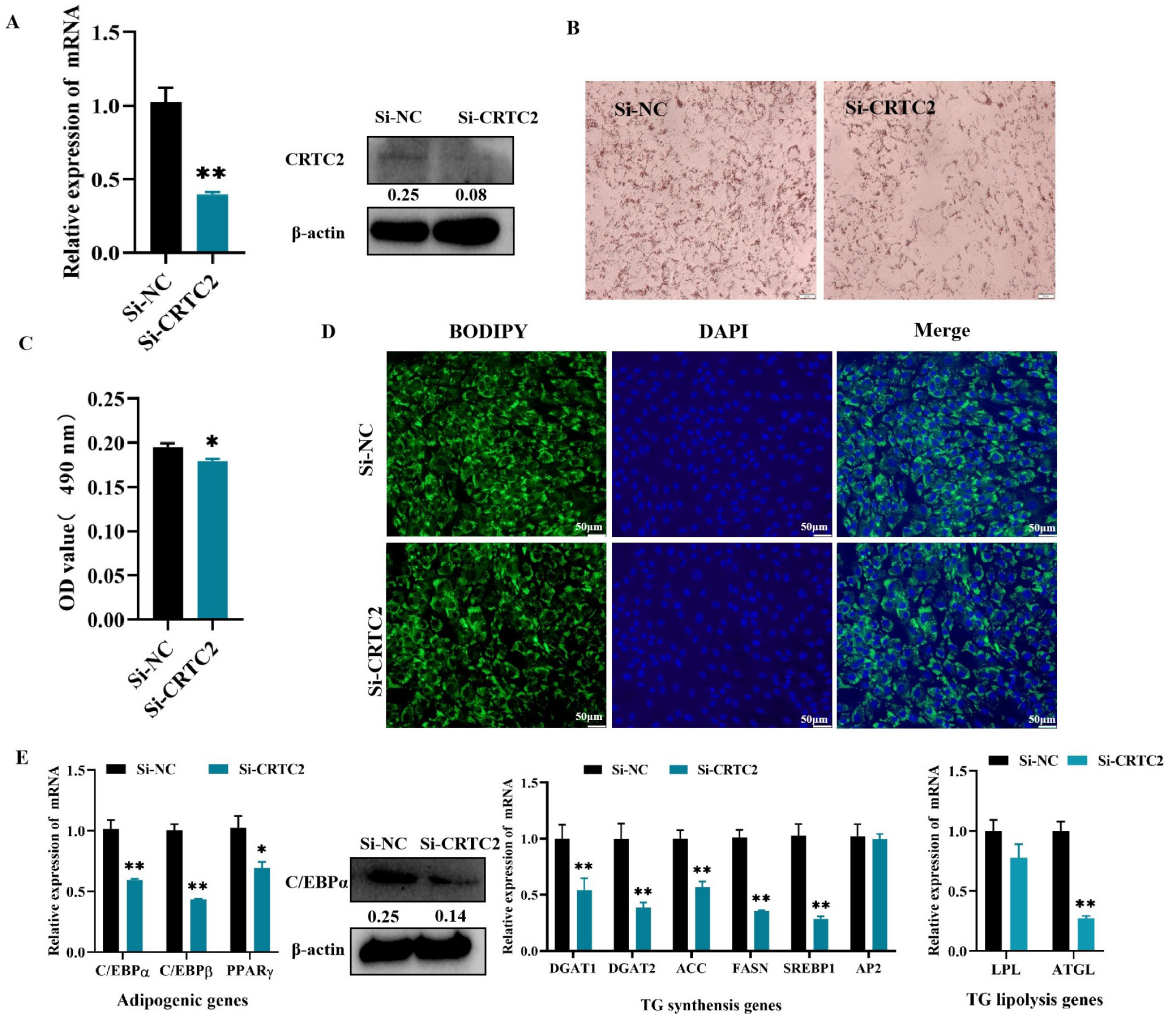
Effect of Si-*CRTC2* on adipocyte differentiation in goat subcutaneous precursor adipocytes. (A) Transfection efficiency of Si-*CRTC2* by qRT-PCR and WB. (B) Oil Red O staining. C: Semi-quantitative evaluation of absorbance assay for Oil Red O content at 490 nm. (D) BODIPY staining. (E) Effects of Si-*CRTC2* on expression of marker genes of lipid metabolism. * p<0.05, ** p<0.01. DAPI, 4′,6-diamidino-2-phenylindole; TG, triglycerides; qRT-PCR, quantitative reverse transcription polymerase chain reaction; WB, Western blot.

To further verify the effect of *CRTC2* on lipogenic differentiation of goat subcutaneous adipocytes, goat subcutaneous precursor adipocytes were transfected with Si-NC or Si-*CRTC2* to inhibit the expression of *CRTC2*. Interference efficiency was measured by qRT-PCR and WB (Figure 5A, Supplement 2). The qRT-PCR data showed that, 48 h after transfection, the interference efficiency of Si-*CRTC2* was greater than 60% compared to the Si-NC group. Oil Red O staining and semi-quantitative and Bodipy staining showed that inhibition of *CRTC2* expression suppressed the accumulation of lipid droplets in goat subcutaneous adipocytes (Figures 5B-5D). In goat subcutaneous precursor adipocytes, Si-*CRTC2* significantly down-regulated the mRNA expression levels of the adipogenic genes *C/EBPα*, *C/EBPβ* (both p<0.01), *PPARγ* (p<*0.05*), and the TG synthesis genes *DGAT1*, *DGAT2*, *ACC*, *FASN*, *SREBP1* and the TG lipolysis gene *ATGL* (p<0.01, Figure 5E, Supplement 2). Taken together, these data further suggest that *CRTC2* promotes lipogenic differentiation of goat subcutaneous precursor adipocytes.

## DISCUSSION

Fat deposition is precisely regulated by many key genes. Therefore, it is important to reveal the genes that affect adipogenesis to promote subcutaneous fat deposition. In this study, we cloned the goat *CRTC2* sequence of 2363 bp, which includes the complete CDS region of 2082 bp. As a member of the CRTC superfamily, deduced *CRTC2* contains three typical features, including the n-terminal CREB-binding domain, the central regulatory domain, and the c-terminal transactivation domain, which are also found in *CRTC2* protein sequences from other mammalian species [23,24].The protein interaction network shows that *CRTC2* may interact with proteins such as *CRTC3*, *AKT2*, *CREB1*, *CREB2*, *SIK1* and *SIK2*. In order to elucidate the function of *CRTC2* in goats, this study utilized qRT-PCR to construct its expression level in various tissues of goats, and the results showed that the expression of *CRTC2* in liver and kidney was significantly higher than that in other tissues. It has been shown that *CRTC2* is the most abundant isoform in liver and pancreatic b-cells and plays an important role in the regulation of gluconeogenesis and cell survival [13].

In this experiment, we further examined the expression of *CRTC2* in goat subcutaneous precursor adipocytes from 0 to 120 h, and found that the expression of *CRTC2* existed throughout the differentiation process and reached the highest expression level at 120 h of induced differentiation. In order to finally elucidate the regulatory role of goat *CRTC2* gene on the differentiation of subcutaneous adipocytes, the present experiments utilized overexpression and interference to study it in depth, and the results of Oil Red O staining and Bobipy staining showed that overexpression of the *CRTC2* gene significantly promoted the accumulation of lipid droplets in intramuscular adipocytes of goats, and the interference of the *CRTC2* gene significantly reduced the accumulation of lipid droplets, which was similar to the results of the study conducted by Han et al [25] in cattle.

To further elucidate its mode of action and molecular mechanism, the overexpression and changes of lipid metabolism-related marker genes in goat adipocytes after interfering with *CRTC2* were examined. It was found that overexpression and interference with *CRTC2* resulted in corresponding changes in the lipid synthesis marker genes *PPARγ*, *C/EBPα* and *C/EBPβ*. Many studies have found that *PPARγ* is a key transcription factor regulating adipocyte development [26], and the expression of *PPARγ* is induced in preadipocytes in response to a variety of factors in the lipogenic induced differentiation medium [27]. *C/EBPα* starts to be expressed at day 4 to 5 of adipocyte induced differentiation [28], while *C/EBPβ* is mainly expressed at the early stage of adipocyte differentiation [29–33]. After overexpression of *CRTC2*, the expression of *C/EBPα* tended to be up-regulated, but not significantly, presumably due to the fact that adipocytes are at the early stage of 2 days of differentiation. Adipose precursor cells undergo changes in cell morphology and a series of related gene expression, and then differentiate into mature adipocytes with increased synthesis of triacylglycerols (TAG) and accumulation of lipids [34]. In this process, *DGAT*1, *DGAT2*, *ACC*, *FASN*, *SREBP1*, and *AP2* are the key fat synthesizing enzymes, while *ATGL* is the key fat synthase. The *FASN*, *SREBP1*, and *AP2* are the key lipogenic enzymes, while *ATGL*, *LPL*, are the key enzymes in lipid synthesis. *ATGL* and *LPL* are related lipolytic enzymes. *DGAT1* shows high expression in the tissues or organs where TAG synthesis is most active [35]. Chen et al [36] found that high expression of *DGAT1* gene increased adipocyte cell size, adiposity, and susceptibility to high-fat diet-induced obesity by constructing transgenic mice overexpressing the *DGAT1* gene in white adipose tissue. Meanwhile *DGAT2* plays a very important role in TAG synthesis and storage [37]. In human (Homo sapiens) body, *FASN* expression is low in most tissues and high in liver, adipose tissue and lactating mammary gland [38]. In this study, the expression of *DGAT1*, *DGAT2* and *FASN* was significantly up-regulated after overexpression of *CRTC2*, and on the contrary, their expression was down-regulated after interference with *CRTC2*. It is hypothesized that there may be a positive regulatory relationship between *CRTC2* and *DGAT1*, *DGAT2*, and *FASN*. *LPL* and *ATGL* likewise play important roles in the process of lipid deposition. The main function of *LPL* is to catalyze the hydrolysis of fatty esters and maintain lipid homeostasis in organisms. When *ATGL* is highly expressed in mice, adiposity is reduced in mice [39]. In this study, after overexpression of *CRTC2*, *HSL* and *ATGL* were significantly up-regulated during the induction and differentiation of subcutaneous precursor adipocytes. The above experimental results suggest that fat synthesis and catabolism are always in a dynamic equilibrium, and that lipolysis occurs at the same time as lipid synthesis, and that fat synthesis and catabolism work together to regulate the deposition of fat [31]. Combining the overexpression and interference results, the goat *CRTC2* gene may promote adipocyte differentiation and influence the process of lipid deposition by up-regulating the expression of *PPARγ*, *C/EBPβ*, *DGAT1*, *DGAT2*, and *FASN*. However, the complete elucidation of the molecular mechanism by which goat *CRTC2* regulates subcutaneous adipocyte differentiation still needs to be further explored.

## CONCLUSION

In this study, the sequence of goat *CRTC2* gene was cloned, which contained the complete CDS region of 2082 bp. The gene is mainly located in the nucleus and encodes a total of 693 amino acids. *CRTC2* gene is mainly expressed in liver and kidney tissues, and the relative expression level is highest after 120 h of inducing lipogenic differentiation of goat subcutaneous adipocytes. Furtherly, we found that the overexpression of *CRTC2* gene promote lipogenesis of goat subcutaneous precursor adipocytes.

## Figures and Tables

**Table 1:** Primer information

Gene name	Forward sequence (5′-3′)	Reverse Sequence (5′-3′)
*CRTC2* (clone)	GTGGCTTGTATTGGGAGGGTG	CGTGCCTTTTCTCATTCTTGG
*CRTC2* (qRT-PCR)	TTCCAATCCGCGTAAGTTTAGT	CACAGCCAATCTGGTTCACAT
*OE-CRTC2*	CCGGAATTCATGGCGACGTCGGGAGCA	CCGCTCGAGCTGAAGCCGGTCACTGCGGAA
*Si-CRTC2*	GUACCUCCAAUUUGACCCATT	UGGGUCAAAUUGGAGGUACTT
*Si-NC*	UUCUCCGAACGUGUCACGUTT	ACGUGACACGUUCGGAGAATT
*UXT*	GCAAGTGGATTTGGGCTGTAAC	ATGGAGTCCTTGGTGAGGTTGT
*C/EBPα*	CTCCGGATCTCAAGACTGCC	CCCCTCATCTTAGACGCACC
*C/EBPβ*	CCGCCTTTAAATCCATGGAA	CTCGTGCTCTCCGATGCTAC
*PPARγ*	AAGCGTCAGGGTTCCACTATG	GAACCTGATGGCGTTATGAGAC
*AP2*	TGAAGTCACTCCAGATGACAGG	TGACACATTCCAGCACCAGC
*SREBP1*	AACATCTGTTGGAGCGAGCA	TCCAGCCATATCCGAACAGC
*FASN*	TGTGCAACTGTGCCCTAG	GTCCTCTGAGCAGCGTGT
*LPL*	GGTGACAGGAATGTATGAGAGTTGG	CCCAAGGCTGTATCCCAAGAG
*ATGL*	CAAGGAGACGACGTGGAACA	CATAGATGTGCGTGGCGTTG
*ACC*	GGAGACAAACAGGGACCATT	ATCAGGGACTGCCGAAAC
*DGAT2*	CATGTACACATTCTGCACCGATT	TGACCTCCTGCCACCTTTCT
*DGAT1*	CCACTGGGACCTGAGGTGTC	GCATCACCACACACCAATTCA

qRT-PCR, quantitative reverse transcription polymerase chain reaction.

**Table 2:** Sequence analysis content and corresponding analysis tools

Analysis content	Software tools used
Looking for open reading frames (ORFs)	NCBI ORF Finder
Amino acid sequence analysis	DNAMAN
Protein physical and chemical property analysis	Protparam
Prediction of functional domain	InterPro
Protein secondary-structure prediction	SOPMA
Protein tertiary-structure prediction	SWISS-MODEL
Phosphorylation site prediction	Netphos
Subcellular localization	PSORT II
Interacting protein prediction	STRING

## SUPPLEMENTARY MATERIAL

**Supplement 1**. Sequencing comparison of overexpressed pEGFP-CRTC2 vectors. **Supplement 2**. A: Gray scale analysis of proteins overexpressing CRTC2.
