# Supplementary material for: Effect of gene CRTC2 on the differentiation of subcutaneous precursor adipocytes in goats
Source: Anim Biosci. 2024 Oct 28;38(5):873–83. doi: 10.5713/ab.24.0248 (PMC12062811; doi:10.5713/ab.24.0248)

Supplementary Figure 1: Sequencing comparison of overexpressed pEGFP-CRTC2 vectors.

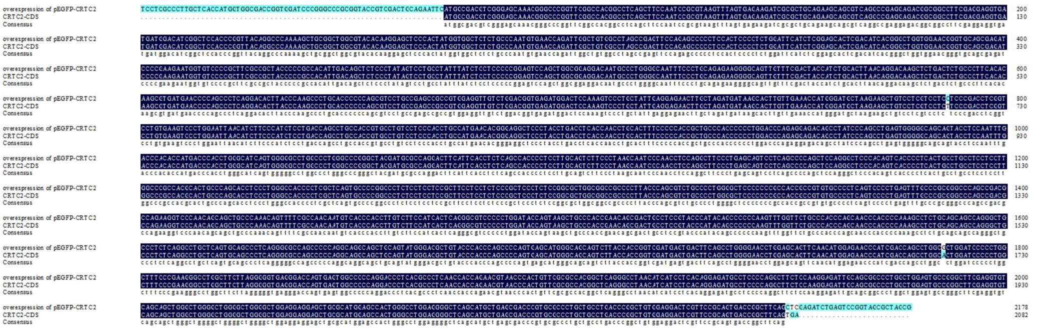

Supplementary Figure 2: A: Gray scale analysis of proteins overexpressing CRTC2. B: Protein grayscale analysis of Si-CRTC2. C: Protein grayscale analysis of the effect of overexpression of CRTC2 on the expression of lipid metabolism marker genes. D: Protein grayscale analysis of the effect of Si-CRTC2 on the expression of lipid metabolism marker genes.

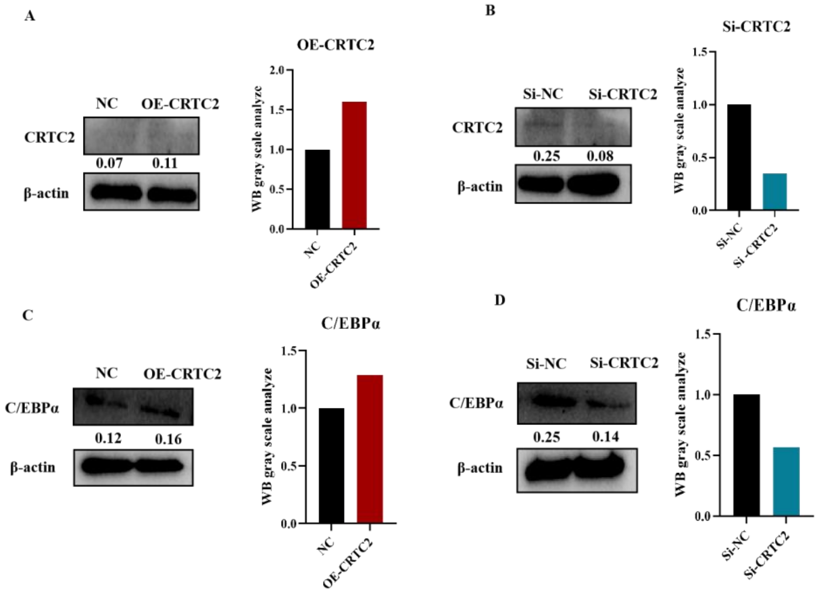

Supplement: Supplementary file 1 [file ab-24-0248-Supplementary-Fig-1,2.pdf]
